# Targeting Microglial α-Synuclein/TLRs/NF-kappaB/NLRP3 Inflammasome Axis in Parkinson’s Disease

**DOI:** 10.3389/fimmu.2021.719807

**Published:** 2021-10-08

**Authors:** Yunna Li, Yun Xia, Sijia Yin, Fang Wan, Junjie Hu, Liang Kou, Yadi Sun, Jiawei Wu, Qiulu Zhou, Jinsha Huang, Nian Xiong, Tao Wang

**Affiliations:** Department of Neurology, Union Hospital, Tongji Medical College, Huazhong University of Science and Technology, Wuhan, China

**Keywords:** Parkinson’s disease, microglia, NLRP3 inflammasome, α-synuclein, neuroinflammation

## Abstract

According to emerging studies, the excessive activation of microglia and the subsequent release of pro-inflammatory cytokines play important roles in the pathogenesis and progression of Parkinson’s disease (PD). However, the exact mechanisms governing chronic neuroinflammation remain elusive. Findings demonstrate an elevated level of NLRP3 inflammasome in activated microglia in the substantia nigra of PD patients. Activated NLRP3 inflammasome aggravates the pathology and accelerates the progression of neurodegenerative diseases. Abnormal protein aggregation of α-synuclein (α-syn), a pathologically relevant protein of PD, were reported to activate the NLRP3 inflammasome of microglia through interaction with toll-like receptors (TLRs). This eventually releases pro-inflammatory cytokines through the translocation of nuclear factor kappa-B (NF-κB) and causes an impairment of mitochondria, thus damaging the dopaminergic neurons. Currently, therapeutic drugs for PD are primarily aimed at providing relief from its clinical symptoms, and there are no well-established strategies to halt or reverse this disease. In this review, we aimed to update existing knowledge on the role of the α-syn/TLRs/NF-κB/NLRP3 inflammasome axis and microglial activation in PD. In addition, this review summarizes recent progress on the α-syn/TLRs/NF-κB/NLRP3 inflammasome axis of microglia as a potential target for PD treatment by inhibiting microglial activation.

## 1 Introduction

Parkinson’s disease (PD) is a chronic progressive neurodegenerative disease associated with aging. Traditionally, PD is pathologically characterized by the loss of dopaminergic neurons in the substantia nigra pars compacta (SNpc) and the widespread aggregation of α-synuclein (α-syn) protein in the form of Lewy bodies and Lewy neuritis (1). Furthermore, brain regions with PD pathology reveal the excessive activation of microglia and elevated pro-inflammatory cytokine levels. Moreover, inflammation in the periphery may be required for the loss of neurons (2), thus indicating inflammation is an important component of PD pathology. The microglia and neuroinflammatory cascade contribute to PD progression, and may occur earlier than the accumulation of α-syn and the onset of PD (3, 4). Thus, targeting inflammatory pathways for PD treatment has gained global attention.

Recent studies have identified the activation of leucine-rich-repeat and pyrin-domain-containing3 (NLRP3) inflammasome and inflammasome adaptor protein apoptosis-associated speck-like protein containing a caspase recruitment domain (ASC) in the SNpc of PD brains (5, 6). In addition, NLRP3 inflammasome and NLRP3-dependent release of inflammatory cytokines are observed in the periphery plasma of patients with PD, indicating the involvement of NLRP3 inflammasome activity in PD pathogenesis (7). NLRP3 inflammasome is a multiprotein inflammatory signaling complex, activated by a variety of microbial or damage-associated molecular patterns (8). Pathogenic protein aggregates, such as α-syn fibrils, another hallmark of PD pathology and the primary component of Lewy bodies, were reported to activate NLRP3 inflammasome in microglia through an interaction with toll-like receptors (TLRs) and the activation of nuclear factor kappa-B(NF-κB) (9, 10). Furthermore, pathological α-syn exerts deleterious effects on synaptic function and mitochondrial homeostasis, in addition to inducing excessive microglial activation (11). This possibly contributes to neuroinflammation and neuronal death (12). In this review, we aimed to focus on the interplay between α-syn pathology and NLRP3 inflammasome, and summarize the role of the α-syn/TLRs/NF-κB/NLRP3 inflammasome axis in the pathogenesis of PD. In addition, we intended to introduce various pharmacological agents or molecular compounds targeting the α-syn/TLRs/NF-κB/NLRP3 inflammasome axis, which might be a promising target for PD treatment.

## 2 Brief Overview of α-syn-TLRs-NF-κB/NLRP3 Axis in PD

### 2.1 Microglial Activation in PD

Microglia are resident mononuclear phagocytes of the central nervous system. They serve as immunological surveillants in healthy individuals, maintaining the homeostasis of the brain microenvironment by shaping neural circuits, fine-tuning synapse plasticity, and interacting with neurons (13). During injury, microglia serve as immune defenders, and are responsible for phagocytosis and the elimination of microbes, dead cells, and protein aggregates by expressing a wide range of immune receptors (14, 15). However, it remains unclear whether they play a beneficial or detrimental role in neurodegenerative diseases, particularly in PD.

Microglial activation and neuroinflammation have received great attention as contributors to PD pathology (16–19). Since activated microglia were firstly reported in the substantia nigra of PD patients post-mortem (20), several studies have described to describing microglial morphological and functional alteration, known as “microgliosis”, in PD patients and animal models (21). In addition, PET scans have demonstrated significantly increased levels of [11C] (R)-PK11195 binding in the pons, basal ganglia, and frontal and temporal cortical regions, thereby indicating the presence of extensive microglial activation. Furthermore, PET imaging reveals early and progressive dopaminergic deficits, and can be used to study the preclinical stages of PD and monitor the progression of the disease (22, 23). A meta-analysis of anti-inflammatory drugs used in clinical trials revealed a negative association between the use of non-steroidal anti-inflammatory drugs (NSAIDs) with the risk of PD progression (24). Epidemiological studies further support the hypothesis that NSAIDs may reduce the risk of developing PD (25), thus suggesting neuroinflammation is involved in the progression of PD pathology. However, there is currently no evidence for the use of NSAIDs for the secondary prevention of PD.

Interestingly, microglial activation occurs and persists in the early stages of PD (26). A detailed study of early and late microgliosis in Thy-1 wild-type α-syn mice revealed that microgliosis initially occurs in the striatum, followed by the SNpc, which precedes dyskinesia. This highlights the susceptibility of dopaminergic neurons (27). Moreover, early microgliosis is associated with α-syn accumulation and neuronal dysfunction (28). A recently published study on transplanting healthy embryonic dopaminergic neurons into the striatum of patients with advanced PD reported on activated microglia in each graft deposit at each tested time point. However, the appearance of pathological α-syn and Lewy bodies in healthy embryonic dopaminergic neurons occurred much later than that of activated microglia (3). In summary, microglial activation is an early event, and may play a key role in pathological α-syn aggregation and α-syn transmission. However, the molecular and regulatory mechanisms involved in microglial activation have not been completely elucidated.

### 2.2 Aggregation and Release of α-syn

**Figure 1:**
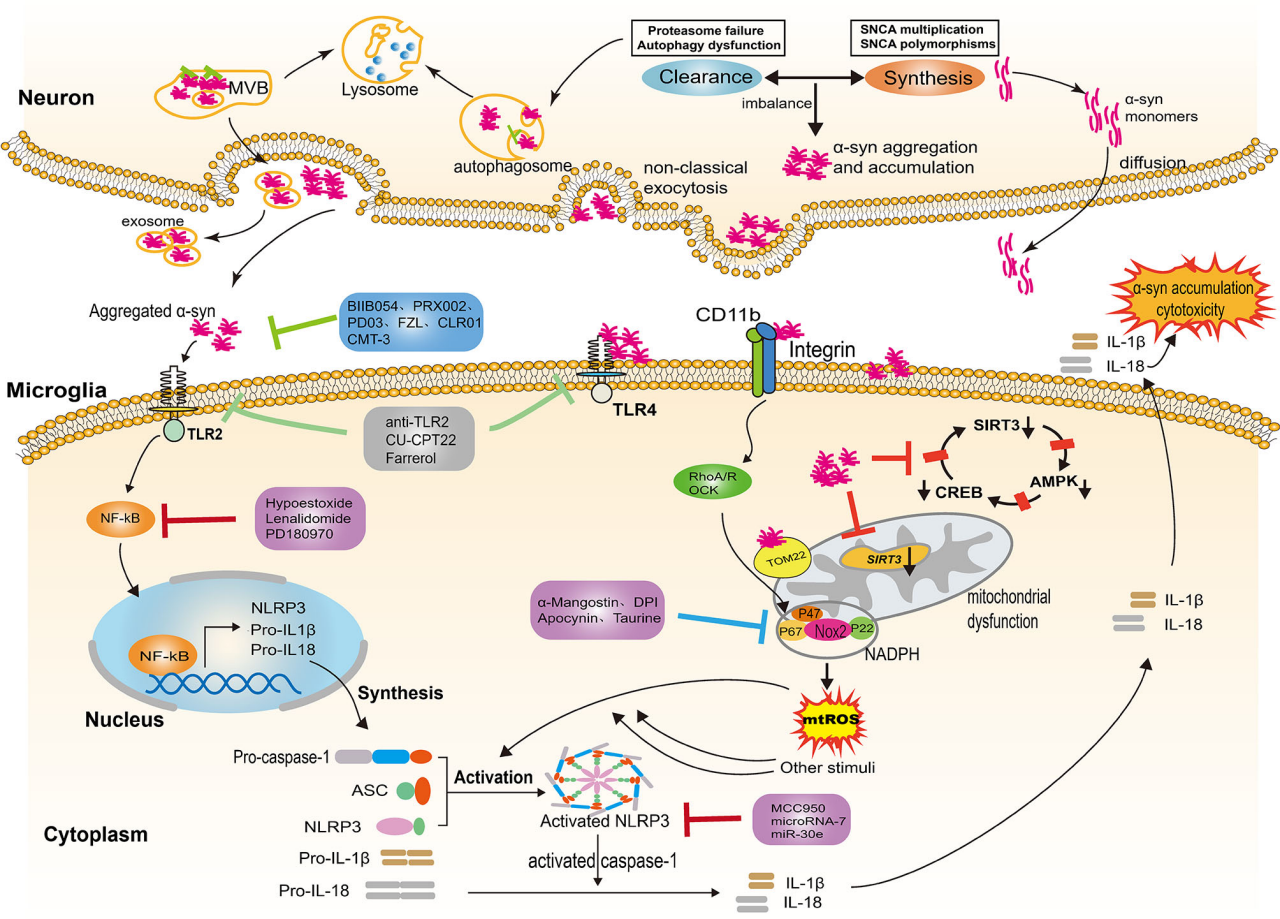
NLRP3 inflammasome activation evoked by α-syn aggregates. In Parkinson’s disease, the imbalance between the α-syn synthesis and clearance contributes to the aggregation and accumulation of α-syn in the neurons. Neurons release α-syn in distinct ways, including passive and active mechanisms; the type of release depends on the different forms of α-syn. Exosomes are also involved in the release of aggregated α-syn. The α-syn aggregates released into the extracellular space provide the priming signal for the activation of NLRP3 through binding to TLR2, triggering NF-κB-dependent upregulation of NLRP3 and production of pro-inflammatory cytokine. In addition, α-syn aggregates impair mitochondrial function following the internalization of α-syn fibrils by microglia, thereby inducing the generation of mtDNA and mtROS. Several molecular mechanisms have been proposed for mitochondrial dysfunction, including the reduction of SIRT3 *via* the AMPKα-CREB signaling pathway, the blockage of TOM20 and the engagement of CD11b. The aforementioned process provides the second “activation” signal for the activation of the NLRP3 inflammasome, which induces activated caspase-1-mediated release of mature IL-1β and IL-18. The inflammatory cytokines are capable of augmenting cytotoxicity and α-syn accumulation. Several therapeutic approaches exert a neuroprotection effect by targeting the α-syn/TLRs/NF-κB/NLRP3 process, including immune antibodies, molecular compounds, the repurpose of pre-existing drugs and natural extracts. NLRP3, leucine-rich repeat- and pyrin domain-containing 3; TLRs, toll like receptors; IL-1β, interleukin-1β; IL-18, interleukin-18; NF-κB, nuclear factor kappa light chain enhancer of activated B cells; TOM20, the translocase of the outer membrane receptors; CD11b, the α chain of a non-covalently associated heterodimeric transmembrane receptor integrin αMβ2; ASC, apoptosis-associated speck-like protein; SIRT3, nicotinamide adenine dinucleotide-dependent deacetylase.

In the central nervous system (CNS), α-syn is mainly located at the presynaptic terminal. Although the physiological functions of α-syn are not fully understood, several studies have shown that it is involved in the regulation of synaptic function and plasticity, vesicle transport, and neurotransmitter release (29, 30). While it is generally accepted that α-syn exists as a monomer in its native state, when interacting with other proteins or under some specific stress-induced conditions, α-syn may aggregate or undergo conformational changes (31, 32). Lewy bodies are one of the pathological features of PD, mainly composed of α-syn aggregates. A number of studies have shown that point mutations of SNCA (33–39), as well as genomic duplications or triplications containing α-syn locus, can enhance the expression of pathogenic α-syn, contributing to α-syn aggregation and accumulation (40, 41). In addition, the imbalance between the synthesis and clearance of α-syn accounts for further aggregation of α-syn (42). Degradation or clearance of α-syn in the cytoplasm is mainly undertaken by the ubiquitin-proteasome system and autophagy-lysosome pathway(see **Figure 1**). The dysfunction of these degradation pathways will lead to aggregation of α-syn, which will eventually contribute to the pathogenesis of PD (43, 44). Excessive aggregation of α-syn or failure to clear it from the cell will result in its secretion and release, which can result in neuron toxicity and α-syn propagation.

Neurons can release α-syn in distinct ways, including passive and active mechanisms; the type of release depends on the different forms of α-syn. Monomers of α-syn, rather than oligomers or aggregates, can diffuse through the cell membrane and are released through compromised cell membranes (45, 46). Aggregated α-syn that has not been degraded is mainly released into the extracellular space through non-classical exocytosis or multivesicular bodies (MVB) (see **Figure 1**) (47). Exosomes derived from the plasma and the CSF of PD patients contain monomers, oligomers, and aggregates, indicating that exosomes are also involved in the release and transfer of aggregated α-syn (48, 49). The α-syn proteins released into extracellular space can be degraded by proteolytic enzymes or taken up by other cells. Neurons, astrocytes, and microglia can all take up α-syn; microglia, as the monitors of the CNS, most easily take up and degrade this protein. After internalization, α-syn can also activate microglia and cause neuroinflammation (50). The NLRP3 inflammasome is involved in the activation of microglia and the release of inflammatory factors.

### 2.3 Structure, Activation and Regulation of NLRP3 Inflammasome

Inflammasomes are multi-protein complexes composed of the following three components: (1) a pattern recognition receptor acting as the sensor molecule, (2) an adaptor apoptosis speck protein (ASC), and (3) pro-caspase-1 as the effector molecule. NLRP3 inflammasome, a well-characterized sensor molecule, can be activated upon a range of acute and chronic inflammatory conditions or stress stimuli (51, 52). In most cell types, the activation of NLRP3 inflammasomes consists of a two-step process. The first assembly signal results in the transcription of the NLRP3 gene by providing an initiation signal. Pattern recognition receptors (including TLRs), tumor necrosis factor 1 (TNFR1) and TNFR2, and nucleotide-binding oligomerization domain-containing protein 2, are involved in this process which leads to the activation of downstream NF-КB and other possible inflammatory transcription factors. The second signal is provided by certain pathogen-associated molecular patterns (PAMPs) and damage-associated molecular patterns (DAMPs), which activate multiple signaling pathways including ion redistribution, lysosomal and metabolic disorders, and mitochondrial dysfunction, where potassium efflux is considered to be the predominant activation mechanism (53, 54). Moreover, it has been reported that NLRP3 can be activated in a non-classical or alternative pathway. For example, cytoplasmic mouse caspase 11 (human orthologs caspase-4/5) can directly sense lipopolysaccharides (LPS) and then induce the cleavage of gasdermin D (GSDMD), thereby forming pores in the membrane, which causes potassium efflux and autocrine activation of NLRP3 inflammasomes (55, 56). Of note, in human or porcine monocytes, TLR4 directly senses LPS and then induces the downstream RIPK1-FADD-caspase-8 pathway to activate NLRP3 inflammasomes (57).

Activated NLRP3 inflammasomes recruits the ASC, leading to the formation of mature caspase-1, thereby inducing the maturation and release of pro-IL-1ß (pro-interleukin-1ß) and IL-18 (interleukin-18). In addition, the activation of NLRP3 can also trigger pyroptosis. This is a kind of GSDMD-dependent programmed inflammatory cell death, which differs from apoptosis and necroptosis (55, 58, 59). NLRP3 activation induces the cleavage of GSDMD upon activated caspase-1 into its functional active form which can insert into the plasma membrane and form pores, leading to cell swelling, membrane rupture, and the further release of IL-1ß and IL-18 (60, 61). Of note, several studies demonstrate caspase4/5/11 can also act on GSDMD and induce pyroptosis through directly sensing LPS, which is so-called caspase-1-independent pyroptosis (56, 62, 63).

#### 2.3.1 The Activation of NLRP3 Inflammasome in PD

Amounting evidence have revealed that NLRP3 inflammasome is supposedly linked to the activated microglia-mediated inflammatory response in PD (10, 64–66). The olfactory bulbs in chronic MPTP-treated mice showed significantly increased levels of IL-1β, caspase-1, as well as activated NLRP3 that were associated with neuroinflammation. Decreased tyrosine hydroxylase (TH) protein levels and a significant increase in abnormal α-syn levels were also observed in olfactory bulb, striatum and SNpc (67). In another study of subacute MPTP-treated mice, NLRP3 deficiency significantly reduced motor dysfunctions, dopaminergic neurodegeneration, and abolished microglial recruitment in SN (68). The assembly of the NLRP3 complex in immune cells upon α-syn-induced stimulation can trigger caspase-1 activation and caspase-1–dependent release of IL-1 and IL-18 (69). Cytosolic NLRP3 inflammasome and key NLRP3 components, including cleaved caspase-1 (p20) and ASC were found elevated in the reactive microglia of post-mortem brain tissue of patients with PD and in the midbrain of α-syn-overexpressed mice (6, 69). Furthermore, neuroinflammatory cytokines produced by activated microglia, such as TGF-α, IL-1β, and IL-6 are elevated in the striatum and CSF of PD patients. IL-1β, a critical neuroinflammatory cytokine, plays a detrimental role in mediating the initiation and propagation of neuroinflammation and pathogenic α-syn. A recent publication revealed the pivotal role of IL-1β expression in the induction and propagation of α-syn aggregation through an IL-1β/IL-1R1-dependent manner. In addition, activated microglia have been associated with reduced autophagy, further contributing to α-syn aggregation. The deletion of IL-1R1 inhibited the transmission of α-syn and TH-positive cell loss (70). NLRP3 activation promotes the secretion of the inflammatory cytokine interleukin-1β/18 (IL-1β/18) and induces pyroptosis, a type of cell death that possesses the potential for inflammation, to rupture microglia to further release IL-1β (71). Furthermore, a detailed study on the transmission of α-syn *via* microglial exosomes highlighted an increase of α-syn aggregation in neurons, when combined with microglial proinflammatory cytokines (72). Inflammatory cytokine has the potential to induce dopaminergic cytotoxicity and exacerbate α-syn aggregation, thus contributing to PD pathology. In other words, NLRP3 inflammasome-mediated release of pro-inflammatory mediators IL-1β and IL-18 partly indicates the role of chronic microglial activation in dopaminergic neuronal death (73).

On the other hand, mitochondrial dysfunction can partly explain the mechanism of NLRP3 activation in PD. A previous study revealed that mitochondrial dysfunction can enhance the activity of the NLRP3 inflammasomes (64). In addition, the production of mitochondrial reactive oxygen species (mtROS) and dysregulated mitophagy are the key regulators of NLRP3 activation (71, 74). Furthermore, a recently published study showed that mitophagy can inhibit the activation of NLRP3 in microglia in a PD model by disrupting mitochondrial clearance, thereby reducing inflammation and improving neuronal loss (65). As an abnormal protein aggregate, α-syn aggregates, acting as one kind of the DAMP, can also partly explain the activation of NLRP3. This process will be elucidated in detail in the following content.

### 2.4 The Relationship Between TLRs, α-syn Forms and NLRP3

TLRs are pattern recognition receptors located on the cell membrane that can induce a series of signal cascades to activate an immune response to stimulation *in vivo* and *in vitro* after recognition of PAMPs and DAMPs (75). Microglia can express all members of the TLR family, with TLR2 and TLR4 being the most widely studied (76). The pathological α-syn released by neurons, as an endogenous DAMPs, can act as ligands of TLR2, and then are transported to lysosomes for degradation or participate in the spread of pathogenic α-syn (77, 78). Studies have found that in TLR2-/-mice, α-syn accumulation in neurons is reduced, accompanied by a decrease in microgliosis and astrogliosis (77). The α-syn released by neurons can also be taken up by astrocytes through TLR4, causing the release of inflammatory factors and further promoting the activation of microglia (79). TLR4 on the surface of microglia are involved in the phagocytosis of α-syn, because TLR4 ablation cause damaged phagocytosis of α-syn (80),and the absence of TLR4 leads to the inhibition of inflammasome activation (81).

Endogenous α-syn can activate microglia by binding to TLR2, but this activation is conformation-dependent, because only the oligomeric, but not monomeric or dimeric forms, acts as a TLR2 agonist able to lead to the release of inflammatory factors (82). However, another study in primary human monocytes suggested that both monomeric and fibrillar α-syn can activate TLR2 (10). Monomers, oligomers, and fibrillar forms may all have the potential to trigger TLR2 located on the surface of microglia, but studies have shown that only fibrillar α-syn aggregates can participate in the traditional NLRP3 activation pathway in microglia and involving the participation of TLR2 (10). Interestingly, the oligomeric form of α-syn has also been reported to interact with TLR1/2 heterodimers to cause nuclear translocation of the p65 NF-КB subunit, thereby activating microglia (83). In summary, α-syn aggregates can activate NLRP3 inflammasomes through canonical pathways, and both TLR2 and TLR4 are involved in this process, which will be explained in detail later. However, it is not clear whether monomers and oligomers can activate NLRP3 in microglia and whether α-syn aggregates can activate NLRP3 through non- canonical or alternative pathways. Further research is needed to explore the exact mechanism by which different forms of α-syn activate NLRP3.

### 2.5 The Importance of α-syn-TLRs-NF-κB/NLRP3 Inflammasome Axis in PD

NLRP3 activation requires the first “assembly” signal and the second “activation” signal. The α-syn protein, an important hallmark of PD, not only serve as the first signal to initiate the assembly of NLRP3 but also as the second signal to activate the NLRP3 inflammasome.

#### 2.5.1 The Interaction Between α-syn and TLR2/NF-κB Serves as the First Signal of NLRP3 Activation

A cross-sectional study reported a linear correlation between the phosphorylated α-syn (P-α-syn) levels and increased IL-1β and NLRP3 levels in PD serum (84). Identifying the association between the aggregation of α-syn and the activation of microglial NLRP3 inflammasome may facilitate revealing the pathophysiology of PD (7). NLRP3 inflammasome has a two-step activation mechanism as follows: (i) “priming,” which induces the transcription of pro–IL-1β and NLRP3 and (ii) “activation,” which involves the assembly of a functional inflammasome complex, following uptake of a pathogen or danger-associated molecular pattern (74). The α-syn aggregates provides the first signal for NLRP3 activation by binding to TLR2, which then induces the nuclear translocation of downstream NF-κB, thereby leading to the production of NLRP3, pro-IL-1β, and pro-IL-18 (85).

TLRs play an indispensable role in α-syn-mediated microglial activation. Watson and colleagues reported that Thy1 α-syn transgenic mice express different types of TLRs, depending on the progression of α-syn pathology and neurodegeneration (27). The activation of TLR2 results in the accumulation of α-syn aggregates in neurons because of the inhibition of autophagic activity (78). In contrast, the inactivation of TLR2 with an antagonist results in autophagy activation and an increased clearance of neuronal α-syn, thereby lowering neurodegeneration (77). These findings reveal a strong association between α-syn aggregates and TLR2 activation. The α-syn oligomeric released by neurons is an endogenous agonist of the microglial surface receptor TLR2 (82). Distinct forms of α-syn have the potential to trigger TLR2 located on the surface of microglia, thereby leading to the nuclear translocation of NF-κB (83). However, only α-syn aggregates can activate the NLRP3 inflammasome, subsequently inducing the caspase-1-mediated release of mature IL-1β and IL-18 (64). Conformation dependence of TLR2 agonists may or at least partially elucidate the aforementioned phenomenon (82). The peculiarity of fibrillated proteins is correlated to their intrinsic structure, most likely their cross-β structure (9). Likewise, TLR2-mediated microglial activation is neurotoxic, and TLR2 ablation or immune antagonism can modulate α-syn transmission and neuroinflammation (77, 86, 87). The above-mentioned studies are predominantly based on animal models. Recently, α-syn was reported to activate NLRP3 in human primary microglia, and induce NLRP3-dependent caspase-1-mediated secretion of IL-1β (88). Furthermore, α-syn aggregates activated NLRP3 by binding to TLR2, but not TLR4 in human induced pluripotent stem cell (hiPSC)-derived microglia (hiMG). Neutralizing TLR2 with neutralizing antibodies almost completely inhibited IL-1β release, and reduced IL-6 and TNF-α release, following α-syn aggregation-induced activation (89). However, similar results were not observed after neutralizing TLR4. Taken together, the interaction between aggregated α-syn and TLR2 support as the first “priming” signal of NLRP3 inflammasome activation.

#### 2.5.2 Microglia Phagocytize α-syn Through TLR4, Acting as the Second Signal for NLRP3 Activation

Apart from TLR2, TLR4 was reported to interact with α-syn, and was involved in microglial activation. Stefanova and co-workers firstly reported that TLR4 absence in microglia was accompanied by a decrease in phagocytic ability, thereby suggesting a close association between the phagocytosis of α-syn and TLR4 (80). Microglia can engulf neuron-released α-syn, which requires the presence of microglial TLR4 (50). Furthermore, it increases the production and secretion of pro-inflammatory cytokines following phagocytosis of α-syn fibrils, thus inducing apoptotic cell death and accelerating the progression of PD (90–92). Similarly, following lentiviral stereotactic injection into the SN of rats, the α-syn species caused severe dopaminergic loss and the production of cellular ROS and severe dopaminergic loss (93). In contrast, TLR4 antagonists substantially reduced ROS and cell death in primary neuronal cultures. Therefore, an indirect inflammatory mechanism involving cytokines produced by glial cells considerably contributes to neuronal death (94). Microglia-induced detrimental effects following α-syn phagocytosis may be related to the NLRP3 inflammasome. This is because TLR4 downregulation can reduce the activation of NLRP3 inflammasomes and the expression of cleaved caspase-1 in PD mice models (81). It is unclear if α-syn-evoked activation of NLRP3 requires the direct engagement of TLR4. Nonetheless, the internalization of α-syn by microglia causes mitochondrial dysfunction, thereby leading to excessive production of mitochondrial DNA (mtDNA) and mitochondrial ROS (mtROS), which directly activate the NLRP3 inflammasome through acting as the second “activation” signal (95, 96).

Several studies have revealed that α-syn fibrils impair mitochondrial function, including the modulation of mitochondrial dynamics-associated protein content and normal mitochondrial morphology in multiple cellular and animal models of PD (97, 98). α-syn-mediated mitochondrial damage produces an overload of mtROS, impairs complex-I-dependent respiration, and decreases mitochondrial membrane potential, thus augmenting inflammatory response and the loss of dopaminergic neurons in PD pathology (95). The mechanism of mitochondrial impairment induced by α-syn fibrils is not well elucidated. However, recently published studies shed light on mitochondrial damage. A previous study reported on decreased mitochondrial SIRT3 protein levels in both rodents overexpressing α-syn and human Lewy body disease brains. Notably, increased α-syn aggregation is correlated with decreased SIRT3 and α-syn-associated decrease in SIRT3 *via* the AMPKα-CREB signaling pathway. Furthermore, SIRT 3 activation attenuates α-syn-induced mtROS and prevents the impairment of mitochondrial dynamics and bioenergetics (99). Another study suggested that oligomeric α-syn binds specifically to TOM20, the translocase of the outer membrane (TOM) receptors, disrupts its normal interaction with the co-receptor TOM22, and inhibits mitochondrial protein import. This eventually leads to an impairment of mitochondrial function, with reduced respiration and excessive mtROS production (100). In addition to producing excessive mtROS through mitochondrial damage, α-syn can also bind to CD11b, the α chain of a non-covalently associated heterodimeric transmembrane receptor integrin α_M_β_2_, and subsequently stimulate the activation of the RhoA-ROCK signaling pathway. This in turn triggers the membrane translocation of nicotinamide adenine dinucleotide phosphate (NADPH) oxidase (NOX2) cytosolic subunit p47phox, thus resulting in NOX2 activation, thereby generating ROS and inducing microglial activation and related neurotoxicity (101).

Various secondary triggers, including adenosine triphosphate, microparticles, and bacterial toxins lead to mitochondrial damage and the release of oxidized mtDNA and mtROS (102). Similarly, α-syn aggregation can also damage mitochondria through a certain mechanism following microglial internalization, thereby producing mtDNA and mtROS, which provide a second trigger for the activation of NLRP3. In α-syn-treated mice, microglia reportedly exhibit a change in mitochondrial morphology, thus indicating fragmented mitochondria. Moreover, mitochondrial dysfunction and mtROS induced by α-syn uptake in microglia contribute to NLRP3 inflammasome activation (85). This finding is consistent with the results observed in hiPSC-derived hiMG that oligomeric/aggregated α-syn induces a significant increase in mtROS generation, a decrease in mitochondrial membrane potential (ΔΨm), and an increase in cytoplasmic mtDNA (89). The aforementioned results may confirm the property of α-syn aggregation to serve as the second “activation” trigger.

In summary, α-syn aggregates provides the first “assembly” signal for the activation of NLRP3 through the TLR2/NF-κB pathway, thus leading to the transcription of NLRP3, pro-IL-1β, and pro-IL-18. In addition, aggregated α-syn impairs mitochondrial function following the internalization of microglia, thereby inducing the generation of mtDNA and mtROS. The aforementioned process provides the second “activation” signal for the activation of the NLRP3 inflammasome, subsequently leading to caspase-1-mediated release of mature IL-1β and IL-18. The inflammatory cytokines augment inflammatory cascades and further accumulation or transmission of pathological α-syn by binding to their receptors or by combining with exosomes containing pathogenic α-syn, thus contributing to neurotoxicity and PD pathology. Therefore, the α-syn-TLRs-NF-κB/NLRP3 axis in activated microglia plays a critical role in neuroinflammation. Targeting NLRP3, particularly the two steps of NLRP3 inflammasome activation evoked by aggregatedα-syn may shed light on PD treatment.

## 3 Targeting α-syn/TLRs/NF-κB/NLRP3 Activation as Therapeutic Targets in PD

### 3.1 Targeting α-syn

Alpha-syn aggregation and transmission are considered responsible for the pathogenesis of PD. Moreover, the aggregated form of α-syn can induce NLPR3 assembly and activation, thus suggesting targeting the aggregation of α-syn may be a feasible approach to prevent α-syn toxicity (103, 104). However, there is inconclusive evidence for these approaches. Researchers have conducted several clinical trials on α-syn in PD, and numerous additional experimental studies aiming at preclinical disaggregation are in progress. Herein, we reviewed several classic drugs that have been tested in clinical trials as well as molecules that reportedly exert neuroprotective effects in rodent animals.

Several clinical trials on anti-α-syn antibodies have been conducted. PD01A and PD03A refer to specific active immunotherapies with synthetic peptides that mimic epitopes on α-syn, thus producing an active substantial humoral immune response by targeting α-syn. Both have been evaluated in phase 1 study in patients with early PD and multiple system atrophy. Furthermore, the repeated administration of PD01A has proven safe and well tolerated over an extended period (105, 106). PRX002, a humanized monoclonal antibody targeting aggregated α-syn, adopts the principle of active immunity. The aforementioned clinical trial aimed to evaluate the efficacy and tolerability of multiple intravenous infusions of PRX002 in patients with early stage PD. It demonstrated robust binding of peripheralα-syn. Moreover, PRX002 concentration in the CSF is likely to engage aggregated α-syn in the brain, thereby exerting a neuroprotective effect by inhibiting the neuron-to-neuron transfer of presumed pathogenic forms of α-syn (107, 108). Other drugs used in clinical trials, including BIIB054 (109, 110), NPT200-11 (111), and MEDI1341 (112) have also made some progress. Despite the progress of some drugs (see **Table 1**) in randomized clinical trials, further studies are needed to evaluate their safety and tolerability, and to determine if targeting aggregated α-syn reduces NLRP3 activation.

**Table 1:** Drugs targeting α-syn tested in clinical trials.

Drug	Mechanism	Primary outcome measures	Reference
PD01A	a specific active immunotherapy with a short peptide formulation	safety and tolerability	(105)
PD03 and Anle138b	a new AFFITOPE^®^ immunotherapy approach	the therapeutic efficacy	(106)
PRX002	humanized monoclonal anti-α-syn antibody,	safety and tolerability	(107, 108)
BIIB054	human-derived monoclonal anti-α-syn antibody	safety, tolerability, and pharmacokinetics	(109, 110)
NPT200−11	α-syn misfolding and aggregation inhibitor	relationship between dose, exposure, and therapeutic benefit pharmacokinetic	(111)
MEDI1341	α-syn antibody	safety, tolerability, and pharmacokinetics	(112)

The inhibition of α-syn aggregation is another feasible strategy that has influenced several groups to concentrate on the disaggregation pathway. Molecular tweezers playing a role in a disordered protein-protein interface have recently received tremendous attention (113). CLR01, molecular tweezers that positively target charged residues of proteins undergoing amyloidogenic processes, decrease α-syn toxicity (114). The neuroprotective and disaggregation effects of CLR01 reportedly have been confirmed in the following cell and animal models of PD. The exposure of axonal terminals to α-syn extracted from patients with PD reduced the aggregation in induced pluripotent stem cell-derived dopaminergic cultures. An improvement in motor defects and decreased α-syn burden were observed in a humanized α-syn overexpressing mouse model (115). In addition, mice injected with α-syn aggregates in the striatum or substantia nigra revealed reduced α-syn-associated pathology (116). The role of neuroprotection and the inhibition of abnormal pathological protein aggregation has been revealed in other neurodegenerative diseases, such as Alzheimer’s disease, multiple system atrophy, and amyotrophic lateral sclerosis (ALS) (117–120). A novel compound (NPT100-18A) reportedly reduces α-syn toxicity and aggregation *via* a novel mechanism that involves displacing α-syn from the membrane by interacting with a C-terminus domain of α-syn. The *de novo* compound exerts a beneficial role in the reduction of α-syn in both neuronal cells and three different α-syn transgenic rodent models (121). **Table 2** summarizes other molecules or compounds aimed at the disaggregation of α-syn, including FLZ (122), CMT-3 (123), aSyn-323.1 (124), hydroxytyrosol (125), NQDA (126), Fasudil (127, 128), and D-520 (129).

**Table 2:** Molecules prevent α-syn aggregation and decrease toxicity.

Molecule	Mechanism	PD model	Effect or outcome	Reference
CLR01	molecular tweezers targeting positively charged residues of proteins undergoing amyloidogenic processes	iPSC-derived dopaminergic cultures treated with PD brain protein extracts; multiple PD mice model	reduced α-syn aggregation in cell somas; improvement in motor defects	(114–116)
NPT100-18A	interact with a domain in the C-terminus of α-syn	*in vitro* cell; mThy1 WT α-syn tg mice; α-syn-GFP tg mice; mutant line of α-syn tg mice	decreased the accumulation of proteinase K-resistant α-syn aggregates; reduced the formation of WT α-syn oligomers	(121)
FLZ	bound to and increase the expression of Hip, a cochaperone of HSP70	α-syn transgenic mice and cells	prevented α-syn aggregation, alleviated motor dysfunction and neuroprotection	(122)
CMT-3	inhibit α-syn amyloid aggregation, disassemble preformed α-syn amyloid fibrils	brain microglial cells	non-toxic and less inflammation on brain microglial cells	(123)
aSyn-323.1, aSyn-336.1, aSyn-338.1	three human anti-α-syn antibodies (isolated from PD patients)	*in vitro* α-syn seeding assay	inhibited α-syn seeding	(124)
hydroxytyrosol	stabilize specific regions of the molecule leading to inhibition of protein fibrillation	SH-SY5Y cells	inhibited α-syn aggregation	(125)
NQDA	two naphthoquinone-dopamine-based hybrid small molecules	SH-SY5Y neuroblastoma cells	inhibited amyloid formation of α-syn; disassembled preformed fibrils of α-syn	(126)
Fasudil	bind directly to tyrosine residues Y133 and Y136 in the C-terminal region of α-Syn	H4 cell culture model; α-Syn(A53T) mice	reduced α-syn aggregation; improved motor and cognitive functions	(127, 128)
D-520	novel multifunctional dopamine agonists promote the disaggregation of both α-syn	*in vitro* assay and an *in vivo* Drosophila synucleinopathy model	neuroprotection; protected fly eyes against the toxicity caused by α-syn	(129)

α-syn antibodies block the interaction between α-syn and TLRs, and mitigate neuroinflammation (130). Similarly, inhibiting α-syn aggregation alleviates cytotoxicity, decreases mitochondrial damage, and ROS generation, consequently exerting a therapeutic effect (131). Anti-α-syn antibodies potently inhibit pathology seeding through the highly selective binding of pathogenic α-syn in a neuronal model of α-synucleinopathy (132). In addition, long-term α-syn knockdown in SN does not cause neurodegeneration or significant functional deficits in dopaminergic projections. Hence, it may be feasible to target α-syn expression in PD (133). Further intense research will shed light on the mechanism underlying the relationship between pathologic α-syn and neuroinflammation in PD.

### 3.2 Inhibition of TLRs

TLRs that can be activated by pathogen-associated molecular patterns [e.g., lipopolysaccharide (LPS)] or damage-associated molecular patterns (e.g., α-syn), and play a profound role in microglial activation. Considering the interaction between aggregated α-syn and TLRs, which subsequently initiates a downstream inflammatory pathway or phagocytosed into the intracellular soma, blocking TLRs may be a specific therapeutic routine for alleviating the toxicity of pathologic α-syn (134). Three major types of therapeutic approaches have been explored, including small molecular compounds, the repurpose of pre-existing drugs, and natural extracts in animal and cell models (see **Table 3**). Anti-TLR2 acts as a functional inhibitory antibody. The neuroprotective effects of anti-TLR2 antibody were observed in *in vitro* studies with neuronal and astroglial cells by blocking neuron-to-neuron and neuron-to-astrocyte α-syn transmission. In an α-syn high expression mouse (α-Syn-tg; under the mThy1 promoter, line 61) administered with anti-TLR2, the latter mitigated α-syn accumulation in neuronal and astroglial cells, neuroinflammation, neurodegeneration, and behavioral deficits (87). The small-molecule compound CU-CPT22 is another heterodimer TLR1/2 (Toll-like receptor 1 and 2) inhibitor. It can reduce the nuclear translocation of NF-κB and the secretion of TNF-α and IL-1β in a MyD88-dependent manner in cultured primary mouse microglia (83). Notably, repurposing candesartan cilexetil, which treats hypertension, can reverse the activated proinflammatory phenotype of primary microglia exposed to pathologic α-syn *via* the inhibition of TLR2.

**Table 3:** Molecules targeting TLRs.

Molecule	Mechanism	PD model	Effect or outcome	Reference
anti-TLR2	a functional inhibitory antibody	PDGFβ-α-syn tg mice stereotaxically injected with TLR2 overexpressing lentivirus; mThy1-α-Syn tg mice administrated with anti-TLR2	alleviated α-syn accumulation, neuroinflammation, neurodegeneration, and behavioral deficits	(87)
CU-CPT22	the small-molecule inhibitor of the heterodimer TLR1/2	cultured primary mouse microglia	reduced secretion of IL-1β, TNF-α, reduced the nuclear translocation of NF-κB	(83)
Candesartan cilexetil	inhibit the expression of TLR2	primary microglia exposed to oligomeric α-syn	reversed the activated proinflammatory phenotype of primary microglia	(83)
Kaempferol	down-regulate the HMGB1/TLR4 pathway	LPS-induced mice	inhibited the production of IL-1β, IL-6, TNF-α; improved striatal neuron injury, and increased the levels of TH and PSD95	(135)
Farrerol	inhibit the TLR4 signaling pathway	BV-2 cells with MPP^+^ treatment	reduction of IL-6, IL-1β, and TNF-α, inhibition of iNOS and the activation of NF-κB	(136)
Schisandrin B	inhibit the interaction between TLR4 and the Toll adapter proteins MyD88, IRAK-1 and TRAF-6	LPS-treated microglia–neuron co-cultures; LPS-treated microglia; ICR mouse	downregulated pro-inflammatory cytokines, including NO, TNF-α, PGE2, IL-1β and IL-; inhibited the production of ROS and NADPH oxidase activity	(137)

COX-2, yclooxygenase-2; TH, tyrosine hydroxylase; PSD95, postsynaptic density protein 95.

Nonetheless, neuroprotection in synucleinopathy through TLR4 blocking does not hold consistent conclusions. Naturally derived compounds have also been explored for their multiple pharmacological properties (e.g., anti-inflammatory and anti-oxidation) in neurodegenerative diseases. Kaempferol (Ka) is a natural extract that inhibits the production of IL-1β, IL-6, and TNF-α, improves striatal neuron injury, and increases levels of tyrosine hydroxylase (TH) by downregulating the HMGB1/TLR4 pathway (135). Farrerol and schisandrin B (see **Table 3**) exert anti-inflammatory effects by suppressing the TLR4 signaling pathway (136, 137). Despite therapeutic strategies aimed at blocking TLRs with some compounds or repurposed drugs have demonstrated neuroprotective effects, further work is required to determine the presence of side effects and if an exposure to toxic α-syn following the blockage of TLRs ameliorates NLRP3 inflammasome. A better understanding of the in-depth mechanism can contribute to the clinical use of the aforementioned compounds.

### 3.3 An Inhibition of Assembly Signal and the Activation Signal of NLRP3 Inflammasome

Microglial activation and neuroinflammation have been proposed as the components of PD. NLRP3 inflammasome activation can partially elucidate microglia-mediated inflammation. Moreover, an increasing number of investigations are aimed at targeting NLRP3 inflammasome-mediated neuroinflammation in activated microglia to find a therapeutic avenue for PD (138). The translocation of NF-κB into the nucleus initiates the transcription of NLRP3 components, pro-IL-1, and pro-IL-18. In addition, mitochondrial impairment and the synthesis of ROS lead to the activation of the NLRP3 inflammasome. α-syn aggregates simultaneously stimulates the two steps of NLRP3 activation. Thus, the negative regulation or blockage of the NF-κB pathway and ROS synthesis may provide an avenue to ameliorate activated NLRP3 inflammasome-mediated neuroinflammation. In addition, specific inhibitors of the NLRP3 inflammasome also exert beneficial effects.

#### 3.3.1 The Inhibition of NF-κB Pathway

Hypoestoxide(HE) has been identified as an NF-κB modulator. mThy1-α-syn transgenic mice that received intraperitoneal injections of HE (5 mg/kg) daily for 4 weeks demonstrated a reduction in pro-inflammatory cytokines released by microglia, an improvement of dopaminergic neuron loss, and motor behavioral deficits. Furthermore, HE significantly decreased the levels of nuclear phosphorylated NF-κB in the frontal cortex of a PD mouse model (139). Interestingly, the repurpose of drugs that were originally used in clinical practice of other systematic diseases can exert a neuroprotective effect in a targeting microglia and neuroinflammation manner. Based on the viable option, lenalidomide, which exerts protective effects on an animal model of ALS, exerts an anti-inflammatory effect in a PD model through the inhibition of NF-κB signaling and subsequent cytokine production (140). Lenalidomide reduced motor deficits and dopaminergic fiber loss in a mThy1-α-syn transgenic mouse model of PD. This protective action was accompanied by a reduction in microgliosis and pro-inflammatory cytokine expression in both striatum and hippocampus. At the molecular level, lenalidomide and thalidomide reduce the activation of NF-κB, TNF-α, IL-6, IL-1β, and IFN-γ expression, together with an increased expression of anti-inflammatory cytokines IL-10 and CX3CL1 (fractalkine) levels in mThy1-α-synuclein transgene mice. The effect of lenalidomide in the mouse microglial cell line BV-2 further confirmed the results obtained *in vivo* (140).

PD180970, a small molecular compound synthesized by chemical biology, reportedly exert an anti-neuroinflammatory effect by inhibiting the release of proinflammatory cytokines, such as IL-6 and monocyte chemoattractant protein-1 through the reduction of NF-κB activation (141). Notably, compared to previously mentioned pharmacological agents that play a neuroprotective role by merely inhibiting the NF-κB pathway, PD180970 inhibits microglia-mediated neuroinflammation. However, it simultaneously enhances the clearance of toxic α-syn aggregates by inducing neuronal autophagy. This is because PD180970 is a small molecule inhibitor of C-Abelson (c-Abl) tyrosine kinase. Patients with PD have elevated tyrosine kinase levels, and their expression is negatively correlated with the levels of pathological protein aggregates (142). Therefore, the inhibition of kinase activity might be a potential target in the treatment of PD (143, 144). PD180970 is capable of ameliorating α-syn mediated toxicity by inducing autophagy in a mammalian target of rapamycin (mTOR)-independent manner in mammalian cells as well as in dopaminergic neurons of the SNpc in the mouse midbrain, thereby diminishing the aggregation of α-syn.

MicroRNAs (miRs) are highly conserved small non-coding RNAs, and are pivotal positive and negative regulators of the inflammatory response (145). In recent years, non-coding RNAs have been identified as a research hotspot because of their strong and complex functions. Specifically, miR-155-5p regulates the macrophage inflammatory response by forming a positive regulatory loop that alters NF-κB activity, and has been extensively characterized (146, 147). Triptolide (T10) is a natural anti-inflammatory and anticancer component isolated from the Chinese herb Tripterygium wilfordii Hook F (148). T10 can protect dopaminergic neurons from inflammation-mediated damage through diverse mechanisms in several PD models (149–151). Moreover, it is capable of suppressing NF-κB activation by regulating the miR155-5p/SHIP1 signaling pathway in fibrillar α-syn-induced primary microglia, thus exerting an anti-inflammatory effect (152). Other small synthetic molecules and naturally derived compounds (see **Table 4**), such as juglanin, KHG26377, calycosin, isobavachalcone, diosgenin, and α-mangostin reportedly exert anti-inflammatory effects by inhibiting the NF-κB pathway in multiple PD models (153–158). However, whether NF-κB inhibition ameliorates NLRP3 activation in these investigations are not well elucidated. Collectively, these results indirectly demonstrate or partially account for the feasibility of previously reviewed agents for the inhibition of NLRP3 inflammasome. This can be attributed to diminished NLRP3-mediated release of pro-inflammatory cytokine IL-1β and mitigated neuroinflammation.

**Table 4:** Molecules targeting NLRP3 and two signals of NLRP3: NF-κB pathway and synthesis of ROS.

Molecule	Mechanism	PD model	Key observation	Reference
Hypoestoxide	NF-κB modulator	mThy1-α-syn transgenic mice	Decreased microgliosis, and pro-inflammatory cytokine gene expression; reduced levels of nuclear phosphorylated NF-κB	(103)
Lenalidomide	Inhibit NF-κB signal pathway	mThy1-α-syn transgenic mice; BV2 microglial cell line	Reduction of TNF-α, IL-6, IL-1β, and IFN-γ expression; Increased the expression of the IL-10; increased CX3CL1 levels	(104)
PD180970	A small-molecule inhibitor of C-Abelson (c-Abl) tyrosine kinase	Co-culture N27 cells with BV2 cell; MPTP-treated mice	Reduction of nitrite release; Reduction in the level of both IL-6 and MCP1	(105)
Triptolide	Suppress NF-κB activity by regulating the MicroRNA155-5p/SHIP1 pathway	Preformed fibrils (PFFs) of human wild-type α-Syn induced primary microglia	Reduction of TNF-α, IL-1β production	(113, 114, 116)
Juglanin	Impede TLR4/NF-κB	LPS-induced mice	Reduction of IL-1β, TNF-α, IL-18 and COX-2	(117)
KHG26377	Suppress NF-κB and MAPK signaling	LPS-stimulated cultured BV-2 microglial cells	Reduced the production of PGE2, TNF-α, IL-1β, ROS, and NO, COX-2, iNOS, TLR4, p-ERK, and p-p38 MAPK	(118)
Calycosin	Suppress the activation of TLR/NF-κB and MAPK pathways	BV2 microglia cells injected with LPS; mice injected with MPTP	Mitigated the behavioral dysfunctions and inflammatory response	(119)
Isobavachalcone	Inhibit the NF-κ B pathway	MPTP mice; LPS-induced BV2 microglia cell line	Reduction of IL-1β, IL-6, TNF-α and NO production	(120)
Diosgenin	Inhibit the TLR/NF-κ B pathway	LPS-induced rat; BV2 microglia cell line	Reduction in mRNA levels of TNF-α, IL-1β and IL-6; Reduction of iNOS, NO and ROS production; Reduction in protein levels of TLR2, TLR4 and nuclear NF-κB	(121)
α-Mangostin	Inhibition of NF-κ B activation; Blockade of NADPH oxidase	α-syn-stimulated primary rat microglial cells; neuron-glia cultures	Reduction of IL-1β, IL-6, and TNF-α production; Reduction of nitrite, iNOS, H2O2, ROS expression	(122)
Diphenyleneiodonium	NADPH oxidase inhibitor	LPS- and MPTP-treated mice; LPS-treated transgenic mice over-expressing human A53T mutant α-syn	Inhibition of the proinflammatory genes TNF-α, IL-β, MHC-II; Inhibition of ROS	(126, 128)
Apocynin	NADPH oxidase inhibitor	Paraquat and maneb-induced mouse PD model	Reduction of ROS production	(131)
Taurine	Inhibit NOX2 activation; Inhibit membrane translocation of p47^phox^	Paraquat and maneb-induced mouse PD model	Reduction in mRNA level of iNOS, TNFα, and IL-1β; Reduction of superoxide production	(135)
microRNA-7	Nlrp3 is a target gene of miR-7	wild type and A53T^tg^/^tg^; Caspase-1 knockout mice; Mesencephalic neuronal and primary microglial cell	suppressed NLRP3 inflammasome activation and protected DA neurons against degeneration	(34)
miR-30e	directly target to Nlrp3	MPTP-treated mice; BV2 cell	suppressed Nlrp3 mRNA and protein expression; attenuated the TNF-α, COX-2, iNOS; decreased Caspase-1 and ASC expressions and IL-18 and IL-1β secretion	(137)
MCC950	small-molecule NLRP3 inhibitor	primary microglia; α-syn PFF–injected mice; 6-OHDA-treated mice; NLRP3 knockout mice	blocked the release of active IL-1β, caspase-1 and ASC; protected against nigrostriatal dopaminergic degeneration	(138)
Kaempferol	inhibit NLRP3 inflammasome activation	LPS-induced PD mouse; A53T^tg/tg^ mice; nlrp3^−/-^ mice	Reduced NLRP3 protein expression; suppressed IL1β secretion; inhibit edCASP1 activation	(140)
FTY720	inhibit NLRP3 inflammasome activation	MPP^+^-treated BV-2 cells and primary microglia MPP^+^-treated mice	reduced the concentrations of TNF-α, IL-1β, and IL-6; inhibited ROS production, attenuated the formation of NLRP3 inflammasome	(141)

MPTP, 1-methyl-4-phenyl-1,2,3,6-tetrahydropyridine; TGF, transforming growth factor; NF-κB, nuclear transcription factor-κB; LPS, lipopolysaccharide; IL, interleukin; NO, nitric oxide; NADPH, nicotinamide adenine dinucleotide phosphate; ROS, reactive oxygen species; iNOS, inducible nitric oxide synthase; TLR, Toll-like receptor; AAV, adeno-associated virus; TNF, tumor necrosis factor; MCP1, monocyte chemoattractant protein-1; NOX2, a NADPH subtype; PGE2, prostaglandin E2, COX2:cyclooxygenase-2; p-ERK, phosphorylated extracellular signal-regulated kinase; p-p38 MAPK, phosphorylated p38 mitogen-activated protein kinase; MyD88, myeloid differentiation factor 88; TRAF6, NF receptor associated factor 6.

#### 3.3.2 ROS Synthesis Blockage

ROS are required for NLRP3 activation (10, 85). Mitochondrial superoxide generation plays a role in NLRP3 inflammasome activation in rotenone-induced and tebufenpyrad-induced PD models (64). The blockage of ROS synthesis may be a feasible option for mitigating NLRP3-mediated neuroinflammation in activated microglia. NADPH plays a critical role in microglial activation, and is the primary source of superoxide generation in microglia (159). NADPH oxidases are membrane-bound, multi-subunit enzyme complexes that transfer electrons across the plasma membrane from NADPH to molecular oxygen. In addition, they generate free radical superoxide and its downstream ROS, by which they participate in multiple cellular activities, such as cellular signaling and post-translational processing of proteins and stress response. According to the new terminology, the NOX family refers to the catalytic subunit of NADPH oxidases, including NOX2 (gp91phox) and its six homologs (NOX1, NOX3, NOX4, NOX5, DUOX1, and DUOX2). NOX2 (gp91phox), the catalytic subunit of NADPH was the first identified and best-characterized member of the NOX family (160, 161). Considering the role of NADPH, inhibition of the activity of NADPH oxidases and its subunit NOX2 can directly decrease the source of ROS, thus ameliorating NLRP3 activation.

Diphenyleneiodonium (DPI), a selective NADPH oxidase inhibitor, can attenuate microglial-mediated neuroinflammation by inhibiting the NADPH pathway. A *in vivo* study has demonstrated that DPI at sub-picomolar concentrations (10^-14^ to 10^-13^M) specifically inhibits NADPH oxidase activation, and protects dopaminergic neurons (162). Nevertheless, this pharmacological agent is not recommended for clinical use at the recommended dose (mg/kg) (163) owing to its non-specificity and high toxicity. However, Wang et al. provided further preclinical evidence to support this therapeutic strategy. In their study, ultra-low-dose DPI not only halted the progression of neurodegeneration and restored motor function but also alleviated α-syn accumulation in multiple PD models (164). In parallel, post-treatment with an ultra-low dose of DPI attenuated LPS-elicited microglia-mediated neuroinflammation and oxidative stress. In addition, mice treated with ultra-low-dose DPI did not demonstrate overt signs of toxicity. The activation of NADPH oxidase is associated with hippocampal and cortical neurodegeneration and non-motor symptoms, particularly cognitive dysfunction. Derived from the medicinal plant Picrorhiza kurroa, apocynin selectively inhibits NADPH oxidase activation *via* metabolic activation by myeloperoxidase (165), and also reduces the membrane translocation of NADPH oxidase cytosolic subunits, thus inactivating NADPH oxidase (166). Apocynin significantly mitigated impairments in spatial learning and memory as well as hippocampal neurodegeneration and α-syn pathology through the suppression of NADPH oxidase in paraquat-and maneb-induced mouse PD models (167).

NOX2, a subunit of NADPH, upregulates the expression in SN of patients with PD and in mouse models. Moreover, extensive preclinical research has demonstrated a crucial role of NOX2 activation in microglia-mediated dopaminergic neurodegeneration. Taurine, a major intracellular free β-amino acid in mammalian tissues, is involved in multiple physiological functions, including neuromodulation, antioxidant, and anti-inflammatory processes (168, 169). In addition, low taurine levels were found in the plasma of such patients, and were negatively associated to motor severity (170). Taurine can block NOX2 activation in paraquat-and maneb-induced mouse PD models, thus potently reduce dopaminergic neurodegeneration and α-syn oligomerization through the suppression of microglial M1 phenotype (171). The inhibition of NADPH oxidase and NOX2 subunit can protect against neuroinflammation and neurodegeneration by decreasing microglial activation, thus indicating the inhibition of ROS synthesis has a beneficial effect However, an alleviation of NLRP3 activation have not yet elucidated. Further studies are required to better understand the role of NLRP3 during diminished ROS synthesis.

### 3.4 NLRP3 Inflammasome Inhibition

Given the contribution of the NLRP3 inflammasome pathway to the pathogenesis of PD. NLRP3 knockout not only protects against nigral dopaminergic degeneration and striatal dopamine deletion but also prevents nigral pathological α-syn formation in PD mice models (5). Targeting NLRP3/caspase-1/IL-1β may be a potential therapeutic strategy. NLRP3 is a target gene of microRNA-7 (miR-7). While transfection with miR-7 inhibits microglial NLRP3 inflammasome activation, anti-miR-7 aggravates inflammasome activation in BV2 cells. Moreover, stereotactic injection of miR-7 mimics into the mouse striatum can protect dopaminergic neurons against degeneration and ameliorate microglial activation in PD mouse models (69). Similarly, the delivery of miR-30e agomir remarkably improved motor behavioral deficits, inhibited the loss of dopamine neurons, and alleviated increased α-syn protein expression in the SNpc of subacute MPTP-induced mice by targeting NLRP3 and suppressing NLRP3 mRNA and protein expression (172). In addition to miRNA, nanomolar doses of MCC950, a small-molecule NLRP3 inhibitor, also exert a neuroprotective effect (6). Furthermore, microglia and bone marrow-derived macrophages from Park2(-/-) and Pink1(-/-) mice are characterized by the exacerbation of NLRP3 inflammasome activation and the enhancement of the caspase 1-dependent release of IL-1β and IL-18. Nevertheless, the aforementioned defect was reversed by MCC950, demonstrating a deficiency of the PD-associated Parkin gene can modulate NLRP3 inflammasome activation (173). Furthermore, the small molecule Kaempferol (Ka) protected mice against LPS- and SNCA-induced neurodegeneration by inhibiting NLRP3 inflammasome activation. Ka reduced cleaved CASP1 expression, and disrupted NLRP3-PYCARD-CASP1 complex assembly with concomitant decreased IL1β secretion *via* the cooperation of ubiquitination and autophagy (174). FTY720 may reduce PD progression by inhibiting NLRP3 inflammasome activation *via* its effects on ROS generation and p65 activation in microglia (175). The inhibition of downstream caspase-1 is another promising target for PD treatment. Codolo et al. inhibited caspase-1 with the specific inhibitor Ac-YVAD-cmk of caspase-1, and subsequently observed a significant decrease in the release of mature IL-1 β from monocytes exposed to fibrillar α-syn. This eventually decreased the susceptibility of dopaminergic neurons (10). Collectively, targeting NLRP3 inflammasome activation is of far-reaching significance, and may act as a novel therapy for PD.

## 4 Conclusion

Aggregated α-syn-and microglia-mediated neuroinflammation play a core role in the onset and progression of PD, respectively. Furthermore, pathological α-syn is closely related to microglia-induced neuroinflammation. This is because the former can initiate the transcription of NLRP3 inflammasome *via* binding to TLR2 and activated NLRP3 inflammasome, following aggregation or fibrillar α-syn phagocytosed by microglia through TLR4, which involves ROS production, thus leading to the latter. The α-syn/TLRs/NF-κB/NLRP3 axis can account for or at least partially explain the mechanism underlying activated microglia-induced neuroinflammation. Thus, targeting the α-syn/TLRs/NF-κB/NLRP3 pathway appears promising as a feasible option to alleviate PD progression. However, these therapeutic agents have limitations in their transformation into clinical use, with regard to the following considerations: First, most cellular or animal models of PD are commonly based on LPS-primed or exogenous MPP+-, MPTP-, 6-OHDA-, paraquat-, or rotenone-induced PD models, which cannot completely mimic the disease progression in patients with PD. Second, the role of the NLRP3 inflammasome and whether NLRP3 inflammasome activation is indeed mitigated is not directly elucidated in most investigations. Third, most of the available α-syn/TLRs/NF-κB/NLRP3 inflammasome modulators are associated with side effects or toxicity, owing to non-specific inhibition and high dose required. Apart from some anti-α-synuclein antibodies, few compounds have been tested for safety and tolerability in clinical trials. However, targeting the α-syn/TLRs/NF-κB/NLRP3 pathway appears promising as a feasible option to alleviate PD progression.

## Author Contributions

The review was designed by YL and YX. Related articles were researched by SY, FW, JSH, LK, JW, YS, QZ, JJH, and NX. The manuscript of this review was prepared by YL and YX. TW critically revised the draft before submission. All authors contributed to the article and approved the submitted version.

## Funding

This work was supported by grants 81974201 and 81671260 from the National Natural Science Foundation of China (to TW) and grants 2017YFC1310200 and 2016YFC1306000 from the National Key R&D Program of China (to TW).

## Conflict of Interest

The authors declare that the research was conducted in the absence of any commercial or financial relationships that could be construed as a potential conflict of interest.

## Publisher’s Note

All claims expressed in this article are solely those of the authors and do not necessarily represent those of their affiliated organizations, or those of the publisher, the editors and the reviewers. Any product that may be evaluated in this article, or claim that may be made by its manufacturer, is not guaranteed or endorsed by the publisher.
